# Novel Probiotic Candidates in Artisanal Feta-Type Kefalonian Cheese: Unveiling a Still-Undisclosed Biodiversity

**DOI:** 10.1007/s12602-024-10239-x

**Published:** 2024-03-13

**Authors:** Iliada K. Lappa, Aikaterini Natsia, Dimitra Alimpoumpa, Electra Stylianopoulou, Ioanna Prapa, Konstantinos Tegopoulos, Chrysoula Pavlatou, George Skavdis, Aikaterini Papadaki, Nikolaos Kopsahelis

**Affiliations:** 1 Department of Food Science and Technology, Ionian University, 28100 Argostoli, Kefalonia Greece; 2 Department of Molecular Biology & Genetics, Democritus University of Thrace, 68100 Alexandroupolis, Greece

**Keywords:** Autochthonous isolates, Lactic acid bacteria, Probiotic properties, White brine cheese, Functional, Sustainable food systems

## Abstract

Autochthonous dairy lactic acid bacteria (LAB) isolates encompass a natural source of starter, adjunct, or probiotic candidates. In this context, traditionally manufactured, using exclusively animal rennet, Feta-type cheeses were collected from five farms located in different regions of Kefalonia island (Greece). The primary objective of this study was to isolate and characterize novel LAB, thereby exploring the unmapped microbial communities of Kefalonian Feta-type cheese and identifying new potential probiotics. The initial screening, included a preliminary gastrointestinal (GI) tolerance assessment (acidic conditions and bile salts), followed by their safety evaluation (hemolytic activity and antibiotic susceptibility). Based on the preliminary screening, selected strains underwent molecular identification and were further investigated for their probiotic attributes (lysozyme and phenol resistance, antimicrobial traits, antidiabetic aspects, cholesterol reduction and adhesion, adhesion to Caco-2 cells, and milk acidification potential). The results showed that 49, out of the 93 retrieved isolates, exhibited resistance to GI conditions, whereas 18 met the safety criteria. The molecular identification revealed strains belonging to the species *Lactiplantibacillus plantarum*, *Limosilactobacillus fermentum*, *Lacticaseibacillus rhamnosus*, and *Lacticaseibacillus paracasei*. The selected rod-shaped 14 isolates displayed a potential probiotic character. The best-performing isolates concerning cholesterol assimilation and adhesion, α-glucosidase inhibition, and epithelial adherence were *Lpb. plantarum* F89, F162, and F254 and *Lcb. paracasei* F214 and F216, whereas *Lcb. paracasei* F70 showed potential as a defined strain starter. The present study explores for the first time the biodiversity of traditionally fermented microbial communities in Kefalonian Feta-type cheese, revealing novel potential probiotic strains that can contribute to the development of innovative functional food products.

**Supplementary Information:**

The online version contains supplementary material available at 10.1007/s12602-024-10239-x.

## Introduction

Among fermented foods, artisanal dairy products have been steadily drawing the attention of customers over the years. Cheese production is closely linked with the geographical characteristics of the production area. Greece, renowned for its mountainous terrain and numerous islands, possesses diverse ecosystems and microclimates that have significantly influenced the evolution of a broad spectrum of cheese-making methodologies with distinct sensory attributes and chemical characteristics [1]. In this respect, the country stands out for its unique cheese varieties that are recognized as protected designations of origin (PDO) and protected geographical indications (PGI), as well as a large number of others that are manufactured in the traditional manner without such registration [2]. Cheese is a prominent product since Greece has often led the globe in respect to the production of sheep milk or holds the second position in the EU for goat milk [3]. Kefalonian white-brine cheese, with a history dating back to Homeric times, is a traditional product of the Ionian Island which also serves as a popular trademark. This artisanal variety of acid-curd cheese is made of a mixture of sheep and goat milk, the latter up to 30%, falling in the family of Feta-type cheese. Total ripening time is usually up to 1 month, and the curds (up to 60% moisture) are subsequently preserved in brine for cold storage. The cheese is made without the use of commercial starter cultures or enzymes, lacking a standardized production process; thus, it displays a strong linkage to regional microclimate. The peculiar taste, texture, and premium nutritional value of this cheese are much appreciated.

The microbial diversity of cheese is highly influenced by traditional methods and ripening time [4, 5]. The dairy farm environment also contributes to the native microbiota of the milk that ultimately affects the cheese [6]. Such a biodiversity identification is of great interest, as it strongly represents the distinctive phenotypes of a certain region. Exploring ecological niches that are preferably uncharted for indigenous cheese microbiomes has opened up new perspectives for revealing dynamic consortia [7]. Artisanal dairy products harbor “wild” lactic acid bacteria (LAB) which can bring novel functional candidates to the forefront of desired technological performance and health-related functionalities. These microorganisms can serve as starters in food fermentations or as adjuncts with multifunctional traits. Literature displays numerous reports on LAB isolates from diverse niches, outlining their potential as probiotic cultures and their usage in food applications [8–12].

So far, this is the first report on the microbiota found in white brine cheese of Kefalonia island. The aim of this study was to explore the indigenous lactic acid bacteria and assess their probiotic potential. Strains with promising characteristics were identified as potential candidates for the development of advanced functional products with a distinct terroir identity.

## Materials and Methods

**Fig. 1:**
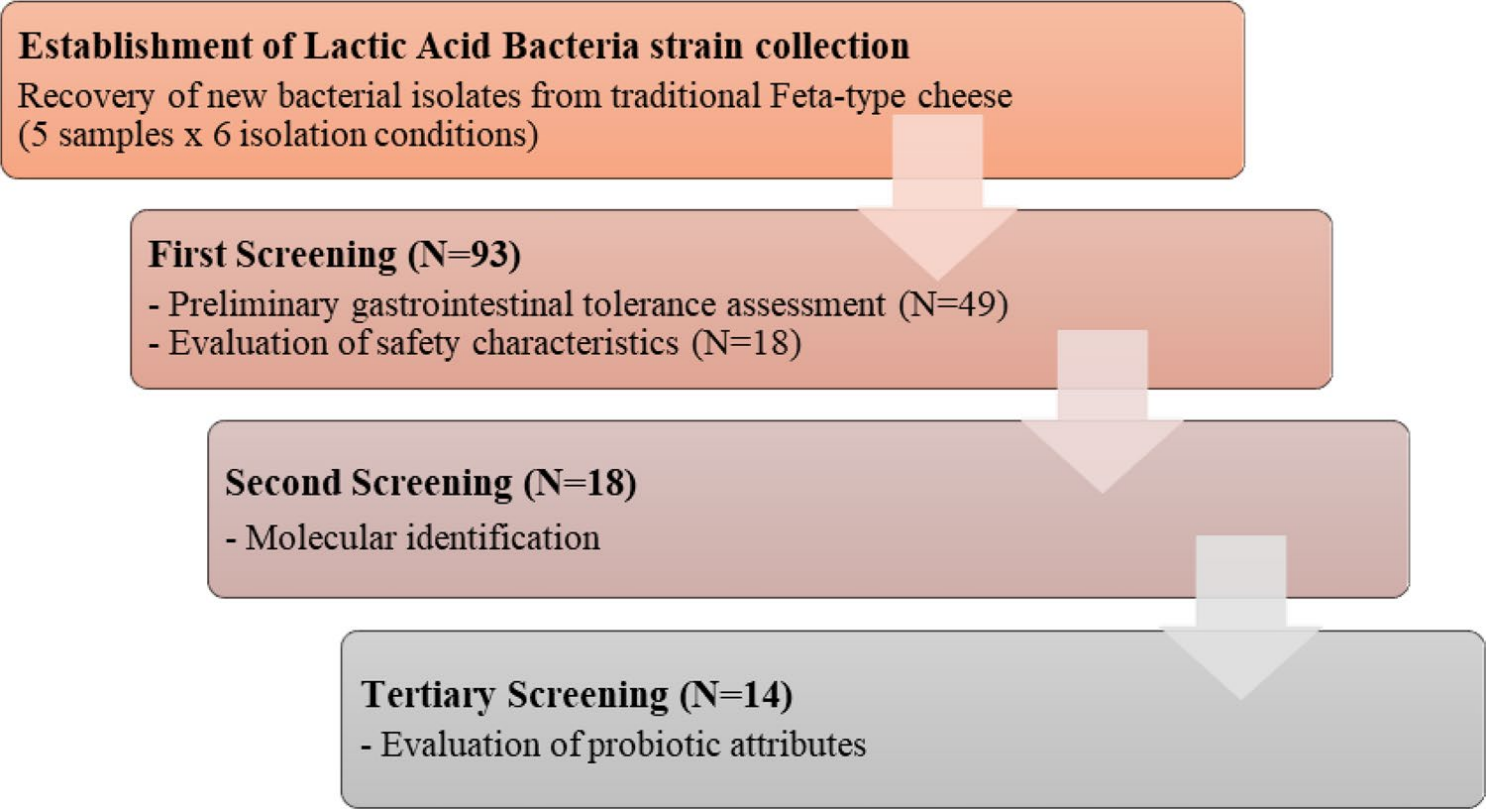
Schematic overview of the study. Three separate screening parts were performed to identify and characterize lactobacilli with potential probiotic properties

Three screening phases were conducted in this study, enabling the identification and characterization of LAB with potential probiotic features. Figure 1 displays the workflow of the employed procedure.

### Microbiological Evaluation of Feta-Type Cheeses

Five white-brine cheese samples were collected from individual local dairy producers in five different geographical regions of Kefalonia island, Greece (Table 1). Cheeses were produced using the traditional method, which does not include the addition of commercial starters or the use of commercial rennet. Samples were collected aseptically and treated by standard microbiological protocols immediately upon arrival to the laboratory in order to determine their microbial profile [7]. In short, 10 g of each sample was homogenized with 90 mL of 2% (w/v) sodium citrate solution, tenfold dilutions were performed and plating was conducted on selective agar media in order to quantify the following microbial groups: (i) total mesophilic bacteria population were enumerated at plate count agar (PCA, Condalab, Madrid, Spain) at 30 °C for 72 h; (ii) mesophilic and thermophilic lactobacilli was enumerated on De Man, Rogosa, and Sharpe (MRS) agar (Condalab, Madrid, Spain) at 22 °C and 42 °C, respectively, for 48 h anaerobically (double agar layer); (iii) mesophilic and thermophilic cocci was enumerated on M17 (Condalab, Madrid, Spain) agar, at 22 °C and 42 °C, respectively, for 48 h; (iv) non-starter lactic acid bacteria (NSLAB) were enumerated on Rogosa agar (Condalab, Madrid, Spain) at 30 °C for 72 h anaerobically (GasPak™, BD, USA); and (v) enterococci were enumerated on kanamycin aesculin azide agar (KAA, Madrid, Spain). Moreover, samples were examined for Enterobacteriaceae after plating on violet red bile glucose agar (VRBGA, Neogen, Lansing, USA) at 37 °C for 24 h and for coliforms on violet red bile lactose agar (VRBL, Neogen, Lansing, USA) at the same conditions. Yeast and molds were counted on yeast extract glucose chloramphenicol (YGC, Condalab, Madrid, Spain) at 25 °C for 3–4 days; coagulase-negative staphylococci were plated on mannitol salt agar (MSA, Condalab, Madrid, Spain) at 37 °C for 24 h, and *Pseudomonas* spp. were cultured on cephaloridine, fucidin, and cetrimide agar (CFC, Condalab, Madrid, Spain) at the same conditions. Samples were also tested for the presence of the pathogenic bacteria *Listeria* spp. and *Salmonella* spp. in compliance with International Organization for Standardization (ISO) methods 11,290–1:2017 and 6579:2002, respectively. All analyses were performed in duplicate for each cheese sample and the results were expressed as log cfu/g.

**Table 1:** Characteristics of cheese samples derived from five different farm locations

Cheese sample ID	Production region	Milk type (%)		Rennin	Storage conditions	pH
		Sheep	Goat			
FC1	Dilinata	70	30	Abomasum	Refrigeration in brine (4.0 months)	4.66 ± 0.13
FC2	Potamianata	75	25	Abomasum	Refrigeration in brine (2.5 months)	3.97 ± 0.08
FC3	Leivathos	80	20	Abomasum	Refrigeration in brine (5.5 months)	4.35 ± 0.12
FC4	Damoulianata	80	20	Abomasum	Refrigeration in brine (5.0 months)	4.29 ± 0.17
FC5	Poulata	80	20	Abomasum	Refrigeration in brine (4.0 months)	4.62 ± 0.11

### Isolation and Phenotypic Characterization of LAB

For the isolation process, five to ten representative colonies were randomly picked out of each countable plate of the two highest serial dilutions of MRS pH 5.5 (22 and 42 °C, anaerobic incubation), MRS (30 °C), M17 (22 and 42 °C), and Rogosa agar (30 °C), in order to generate a highly representative display of the existing biodiversity of each cheese sample. Prior to characterization, isolates were stored in nutrient solutions containing 25% glycerol (v/v) and kept at −80 °C. Selected single colonies were macroscopically examined for distinct morphological dissimilarities (color, size, surface, shape) and purified using two successive streaking of subcultures on the respectful isolation medium. All presumptive LAB were examined for cell morphology by Gram staining and subjected to a catalase production test. The identified Gram-positive rods or cocci were finally transferred to MRS or M17 broth, respectively, containing 25% glycerol (v/v) and stored at −80 °C for further study.

### Preliminary Screening and Safety Assessment of LAB Isolates

#### Acidic pH and Bile Salt Resistance

Stored pure cultures of LAB isolates were reactivated in broth (MRS for rod or M17 for cocci) for 24 and 16 h successively at 37 °C. The resistance of isolates to acidic pH and bile salts’ presence was evaluated as previously described by [13]. Briefly, overnight cell cultures were harvested by centrifugation (12,000 × g, 10 min, 4 °C); washed twice with phosphate buffer saline (PBS), pH 7.2; and inoculated in prepared PBS at pH 2.5. Similarly, bacterial cells were resuspended in PBS solution (pH 8.0) containing 1% (w/v) bile salts (Oxoid, Hampshire, UK). Population resistance to these treatments was assessed after 3 h of incubation at 37 °C. Cultures were withdrawn and the plate count method was used for cell viable count enumeration. The results are expressed as follows:

$$\mathrm{Survival\;rate }\left(\mathrm{\%}\right)= \left(\mathrm{log\;cfu\;A}/\mathrm{log\;cfu\;B}\right)\times 100$$

where log cfu A is the final population of viable cells at 3 h of incubation, and log cfu B is the initial inoculated count.

#### Hemolytic Activity

For assessing hemolytic activity, overnight bacterial culture isolates were streaked on 5% sheep blood agar plates (Biocompare, San Francisco, USA), followed by incubation at 37 °C for 48 h [2]. The hemolysis reaction was characterized by the observation of: greenish or discolored medium surrounding the colony (β-hemolysis) and light-yellow zones around colonies (α-hemolysis). No zones of presence around colonies were designated as γ-hemolysis and the specific isolates were further used since they were considered safe and exhibited no hemolytic activity.

#### Antibiotic Susceptibility

The microdilution-broth assay was performed in order to assess the susceptibility pattern of isolates to common antibiotics. The selected isolates were tested for their resistance to various antibiotics according to the technical guidelines of the ISO [17]. Ampicillin, gentamycin, kanamycin, streptomycin, tetracycline, chloramphenicol, and erythromycin (purchased from Applichem, Spain) were used at concentrations ranging from 0.5 to 1024 mg/L. Each well was filled with 100 mL of diluted inoculum and 100 mL of antibiotic solution using LAB susceptibility test medium (LSM, HiMedia Laboratories, Mumbai, India), and plates were incubated at 37 °C for 48 h. Microorganisms’ growth was visually monitored, and the minimum inhibitory concentration (MIC) value of each antibiotic was determined as the lowest concentration able to inhibit visible cell growth. The MIC cut-off values established by the European Food Safety Authority [18] were taken into consideration in order to classify LAB isolates as susceptible or resistant to each antibiotic that was tested.

### Molecular Identification of LAB Isolates

Microbial DNA was extracted using the NucleoSpin™ Soil kit (Macherey–Nagel, Germany) according to the manufacturer’s instructions, followed by amplification of the hypervariable regions of the *16S rRNA* gene. The amplicons were then subjected to Sanger sequencing by StarSEQ GmbH (Mainz, Germany). The online version of BlastN (National Center for Biotechnology Information, 2021) [14] was employed for the identification of the species group the isolates belong to. For the identification of the strains within the *Lacticaseibacillus casei* group, a specific multiplex PCR targeting the mutL locus was employed [15]. The expected sizes of the amplified products were 253 bp for *Lacticaseibacillus paracasei*, 666 bp for *Lcb. casei*, and 801 bp for *Lacticaseibacillus rhamnosus*. Similarly, a specific multiplex PCR targeting the recA locus was employed for identification within the *Lactiplantibacillus plantarum* group [16]. The expected sizes were 108 bp for *Lactiplantibacillus paraplantarum*, 219 bp for *Lactiplantibacillus pentosus*, and 319 bp for *Lpb. plantarum*.

### Assessment of Probiotic Properties

#### Bile Salt Hydrolase Activity

Bile salt hydrolase (BSH) activity was assessed using a qualitative direct plate assay, as described by Hernández-Gómez et al*.* [19]. Specifically, overnight cultures were streaked onto MRS agar supplemented with 0.5% (w/v) sodium salt of taurodeoxycholic acid (TDCA, T0875, Sigma–Aldrich, St. Louis, MO, USA) and incubated at 37 °C for 48 h anaerobically. The hydrolysis effect was indicated by the formation of deconjugated bile acid precipitation zones as opaque haloes around the colonies, or dissimilar morphologies of the colony surface compared with the control MRS plates (plates without TDCA).

#### Lysozyme Resistance and Phenol Tolerance

LAB were examined in vitro for their ability to withstand the presence of lysozyme (saliva enzyme) as described by Akmal et al*.* [20] slightly modified. In short, freshly prepared cultures were pelleted by centrifugation (12,000 × g, 10 min, 4 °C), washed with PBS, and resuspended in a sterile electrolyte solution of NaHCO_3_ 1.2 g/L, NaCl 6.2 g/L, CaCl_2_ 0.22 g/L, and KCl 2.2 g/L containing 100 μg/mL of lysozyme (Sigma-Aldrich). Samples were incubated at 37 °C and after 2 h, the viable cell count was enumerated by the plate count method. A medium without lysozyme was used as a control for cell growth. Lysozyme resistance was measured as the survival rate of each strain using the following formula:

$$\mathrm{Relative\ growth}\;(\%) = (\mathrm{T/C})\times100$$

where T denotes the growth of sample treatment and C denotes the growth of the control sample.

Phenol tolerance was carried out based on Elbanna et al*.* [21]. The ability of isolates to grow in the presence of phenol was assessed by inoculating 1% of an overnight culture in MRS broth (Condalab, Madrid, Spain) with and without 0.4% phenol. The viability was assessed by plating successive dilutions on MRS agar and incubating anaerobically at 37 °C for 48 h in order to enumerate surviving population expressed in log cfu/mL.

#### Antimicrobial Activity

Antimicrobial activity spectrum was determined using the following indicator pathogen strains: *Listeria monocytogenes* FMCC Β124, *Staphylococcus aureus* FMCC Β134, *Pseudomonas aeruginosa* FMCC Β223, *Penicillium expansum* F1, and *Aspergillus flavus* F7, *Aspergillus niger* F8 (all kindly provided by the Laboratory of Microbiology and Biotechnology, Agricultural University of Athens, Athens, Greece), as well as *Klebsiella oxytoca* 1333, *Staphylococcus epidermidis* 121 and *Clostridium difficile* (kindly provided by the Laboratory of Applied Microbiology and Biotechnology, Democritus University of Thrace, Alexandroupolis, Greece). Bacterial strains were subcultured twice in Tryptic Soy Broth (TSB) (Condalab, Madrid, Spain) for reactivation at 37 °C for 24 h. *Aspergillus* spore suspensions were generated by collecting spores from 7-day-old colonies cultured on Malt Extract Agar (Condalab, Madrid, Spain) at 25 °C in the dark.

LAB inhibition abilities were screened in a turbidimetric assay using the broth microdilution method, as reported by Nelios et al*.* [22]. In short, cell-free supernatants (CFSs) were collected from 24-h LAB cultures propagated twice in MRS broth at 37 °C. Cells were harvested (12,000 × g, 10 min, 4 °C) and CFS pH was neutralized to 6.5 using 5 N NaOH in order to prevent the effect of the cultures’ organic acids. Filter-sterilized (0.22 μm pore size) (Merck, Darmstadt, Germany) CFSs were obtained and stored at −20 °C until use. Neutralized CFSs were used at a concentration of 90% and cultured with diluted, in freshly prepared TSB, pathogen cell suspension (10^5^ cfu/mL) in a 96-well microplate, at a final volume of 200 μL. Plates were incubated at 37 °C for 24 h and spectrophotometrically measured at 0- and 24-h intervals. The growth of pathogens in the presence or absence of CFSs was monitored at 600 nm. Absorbance readings were recorded at two independent experiments using a Multiskan FC microplate photometer (Thermo Fischer Scientific Inc., Waltham, Massachusetts, USA). The effect of CFSs was evaluated as the percentage of growth inhibition and calculated as follows:

$$\mathrm{Growth\ inhibition} \left(\%\right)=\left(1-\left(\mathrm{treatment\ growth/control\ growth}\right)\right)\times 100$$

Antifungal assays were conducted on dual culture experiments according to the procedure outlined by Lappa et al. [23]. Fungal co-cultures were performed in MRS agar where LAB isolates (10^6^ cfu/mL) were inoculated in agar. Following medium solidification, 10 μL of conidia suspension (10^6^ spores/mL) was placed in the center of the agar plate and microorganisms were co-cultured for 3 days at 30 °C. The antagonistic effect of LAB against aspergilli was assessed by measuring the fungal growth according to the following formula:

$$\normalsize \mathrm{Fungal}\;\mathrm{inhibition}\;(\%)=(1-(\mathrm{growth}\;\mathrm{with}\;\mathrm{LAB}/\mathrm{growth}\;\mathrm{without}\;\mathrm{LAB}))\times100$$

#### Inhibition of α-Glycosidase

In order to determine the α-glucosidase inhibitory activity of the wild-type LAB strains, cell-free supernatants from each strain were prepared according to Chen et al*.* [24]. In brief, LAB cultures were grown in MRS broth for 24 h at 37 °C, and then, CFSs were obtained by centrifugation (8000 × g for 20 min, 4 °C). pH was adjusted to 7.4 (using 5 M NaOH) and subsequently, CFSs were sterilized by filtration using a 0.22-μm pore size filter. CFS α-glucosidase inhibitory activity was assessed according to the method described by Nelios et al*.* [22]. Absorbance readings were recorded at 405 nm by SpectraMax ABS Microplate Reader (Molecular Devices LCC, San Jose, California, USA) and α-glucosidase inhibition was calculated according to Nelios et al*.* [22]. The probiotic *L. rhamnosus* GG (ATCC 53103) was used as a reference strain for comparison reasons.

#### In Vitro Cholesterol Removal and Adhesion

LAB isolates were investigated for their ability to assimilate cholesterol using the method described by Wang et al*.* [25], with slight modifications. In brief, a water-soluble form of polyoxyethylene-cholesteryl sebacate cholesterol (PEG 600, Sigma-Aldrich, Missouri, USA) was added to MRS broth at a final concentration of 150 μg/mL and 1 mL of each isolate was inoculated (overnight cultures ca. 1 × 10^9^ cfu/mL) and incubated at 37 °C for 24 h. Uninoculated MRS broth-cholesterol-PEG 600 was used as the control. Following incubation, bacterial suspensions were centrifuged (12,000 × g, 10 min, 4 °C) and the supernatants were collected. The remaining cholesterol concentrations in the supernatants and uninoculated MRS broth were determined by a colorimetric method, as described by Rudel and Morris [26] and Liong and Shah [27]. Briefly, 500 μL of 33% (w/v) KOH (Merck, Darmstadt, Germany) and 1 mL absolute ethanol (Sigma-Aldrich) were added to 500 μL of sample and the solutions were heated in a water bath at 37 °C for 10 min, followed by cooling at room temperature. After cooling, 1.3-mL distilled water and 2 mL hexane (Honeywell International, North Carolina, USA) were added and vortexed for 1 min. Then, the mixtures were allowed to stand for 15 min at room temperature for phase separation. Subsequently, 1 mL of the upper hexane layer was transferred into a glass tube and the solvent was allowed to evaporate overnight. The residue was dissolved in 2 mL o-phthalaldehyde reagent (Merck) (0.05 g o-phthalaldehyde in 100 mL acetic acid). The mixture was kept at room temperature for 10 min. Then, 500 μL of concentrated sulfuric acid was added and the solutions were well mixed. After letting the mixture stand for 10 min, absorbance was determined at 570 nm using a SpectraMax ABS Microplate Reader. A standard curve was generated using the following concentrations: 50, 100, 125, 166.6, 250, and 500 μg/mL cholesterol in MRS broth (*R*^2^ = 0.99), and cholesterol assimilation was determined by the standard curve of the following equation:

$$\mathrm{Cholesterol\ assimilation}\ (\%)=(({\mathrm{C}}_{0}- {\mathrm{C}}_{1})/{\mathrm{C}}_{0}) \times 100$$

where C_0_ and C_1_ represent uninoculated and inoculated MRS broth-cholesterol-PEG 600, respectively*. Lpb. plantarum* DSM 20174 was used as a reference strain for comparison reasons [28]. All treatments were carried out in triplicate.

The capacity of lactobacilli strains to adhere cholesterol was also tested by flow cytometry, according to Bosch et al. [29] with slight modifications. Briefly, a bacterial culture of 10^8^ cells/mL was mixed with the cholesterol chemical analogue CholEsteryl BODIPYTM FL C12 (Invitrogen, Massachusetts, USA) at a final concentration of 5 mg/mL and incubated at 37 °C for 15 h in the dark, with orbital agitation. Cells were harvested by centrifugation (3200 × g, 10 min, 4 °C), washed twice in PBS, and resuspended in PBS at a final volume of 500 μL. Bacterial samples were analyzed on a flow cytometer (Attune NxT flow cytometer, Thermo Fisher Scientific) and data analysis was performed with the FlowJo V10 software (BD Biosciences, Franklin Lakes, New Jersey, USA). Propidium iodide staining (10^3^ mg/mL, Invitrogen) was used to label non-viable cells. The procedure was carried out in triplicate.

#### Bacterial Adhesion to Caco-2 Monolayer

The human colon epithelial cancer cell line Caco-2 (ATCC, Manassas, Virginia, USA) was used as an intestinal epithelial cell model. Caco-2 cells were routinely cultured in Dulbecco’s modified Eagle’s essential (DMEM) high-glucose medium (Biosera, Nuaille, France) supplemented with 20% v/v Fetal Bovine Serum (FBS, Gibco, New York, USA), 2 mM L-glutamine (Biosera), 100 U/mL penicillin (Biosera), and 100 U/mL streptomycin (Biosera) at 37 °C in an atmosphere of 5% CO_2_/95% air. For differentiation, 5 × 10^5^ cells were seeded in each well of a 24-well tissue culture plate (Corning, New York, USA) and cultured in DMEM high-glucose medium supplemented with 10% v/v Fetal Bovine Serum, 2 mM L-glutamine, 100 U/mL penicillin, and 100 U/mL streptomycin for 21 days (the medium was daily changed). Twenty-four hours prior to the adhesion assay, the culture medium of the differentiated Caco-2 monolayer was replaced with antibiotic-free medium.

Bacterial strains were grown in MRS broth at 37 °C for 24 h, harvested by centrifugation (4000 × g, 4 °C, 20 min), washed twice with sterile quarter-strength Ringer’s solution, and resuspended in antibiotic-free DMEM culture medium at a density of 10^8^ cfu/mL. For the adhesion assay, 500 μL of bacterial cell suspension was added on each Caco-2 monolayer of the 24-well tissue culture plate and incubated at 37 °C in a humidified atmosphere of 5% CO_2_ for 2 h. At the end of the incubation, cells were washed twice with PBS and collected using a scraper in 500 μL of sterile quarter-strength Ringer’s solution. Bacterial cell concentration (cfu/mL) was estimated by serial dilutions plated on MRS agar plates and incubated at 37 °C for 72 h. The percentage (%) of microbial cells attached onto the Caco-2 monolayer was estimated as the ratio of the number of bacterial cells that remained attached, to the total number of bacterial cells initially added to the well. The experiments were carried out in triplicate.

### Milk Acidification Assay

In this experiment, the designated isolates were examined for their acidification activity in reconstructed skimmed milk (10% w/v, Condalab, Madrid, Spain) by recording pH level decrease. Specifically, the acidifying ability of the designated isolates was assessed by the addition of 1% fresh overnight culture aliquots of skimmed milk, followed by incubation at 37 °C. pH levels were determined at inoculation time (*t*_0_) and after 6-, 12-, and 18-h intervals, performing two independent experiments.

### Statistical Analysis

The results are displayed as the mean values ± the standard deviation. Significant differences were determined by the analysis of variance (ANOVA), and mean differences were identified using a post hoc test with confidential intervals of 95%.

## Results

### Microbiological Analysis of Cheeses and Isolation of LAB

The microbial population groups of Feta-type cheese samples are summarized in Table 2. More specifically, the dominant population consisted of mesophilic aerobic bacteria ranging from 7.75 to 8.89 log cfu/g and mesophilic and thermophilic cocci ranging from 6.94 to 8.82 log cfu/g. NSLAB levels were recorded from 6.74 to 8.49, while lower counts were recorded for both enterococci (4.45–6.89 log cfu/g). Enterobacteriaceae and coliforms counts were rather low (0.00–4.58 log cfu/g) for samples FC1, FC3, and FC5, while samples FC2 and FC4 did not include any counts. Regarding micrococci, yeast and mold counts ranged from 2.58 to 4.62 log cfu/g. As for the examined pathogens, no presence of *Pseudomonas* spp., *Salmonella* spp., or *L. monocytogenes* was detected in any of the samples, as determined by the ISO techniques [30, 31]. Out of the five cheese sample batches studied, a total of 93 Gram-positive, catalase-negative bacterial isolates were recovered. On the basis of cell morphology, isolates were grouped as 70 rods and 23 cocci.

**Table 2:** Microbial counts corresponding to each cheese sample collected, from five different farms

Presumptive microbial group	FC1	FC2	FC3	FC4	FC5
Total mesophilic bacteria	8.64 ± 0.07	8.89 ± 0.05	8.18 ± 0.06	7.75 ± 0.06	7.79 ± 0.02
Thermophilic lactobacilli	6.28 ± 0.15	8.10 ± 0.95	8.09 ± 0.06	8.09 ± 0.06	7.79 ± 0.03
Mesophilic lactobacilli	7.50 ± 0.20	7.80 ± 0.03	7.99 ± 0.01	7.76 ± 0.01	7.59 ± 0.05
Thermophilic cocci (presumptive lactococci)	7.90 ± 0.06	8.40 ± 0.06	7.17 ± 0.12	6.94 ± 0.02	7.26 ± 0.19
Mesophilic cocci (presumptive streptococci)	7.37 ± 0.28	8.82 ± 0.05	7.90 ± 0.07	7.90 ± 0.07	7.65 ± 0.11
NSLAB	7.44 ± 0.14	8.49 ± 0.01	6.74 ± 0.20	6.75 ± 0.07	6.74 ± 0.02
Enterococci	6.20 ± 0.13	6.13 ± 0.02	6.89 ± 0.11	6.22 ± 0.82	4.45 ± 0.21
Enterobacteriaceae	2.49 ± 0.06	Below DL^a^	4.58 ± 0.22	Below DL	2.33 ± 0.04
Coliforms	2.45 ± 0.05	Below DL	3.61 ± 0.07	Below DL	Below DL
Micrococci	3.70 ± 0.10	3.53 ± 0.02	3.43 ± 0.02	3.62 ± 0.16	3.48 ± 0.16
Yeasts and molds	2.58 ± 0.15	4.62 ± 0.01	3.39 ± 0.01	4.39 ± 0.43	3.68 ± 0.02
*Pseudomonas* spp.	nd^b^	nd	nd	nd	nd
*Salmonella* spp.	nd	nd	nd	nd	nd
*Listeria* spp.	nd	nd	nd	nd	nd

^a^*DL* detection limit: DL was 2.0 log cfu/g for Enterobacteriaceae and coliforms (pouring inoculation method)

^b^*nd* not detected

### Preliminary GI Tolerance Assessment

#### Acid and Bile Salt Tolerance Profile

**Fig. 2:**
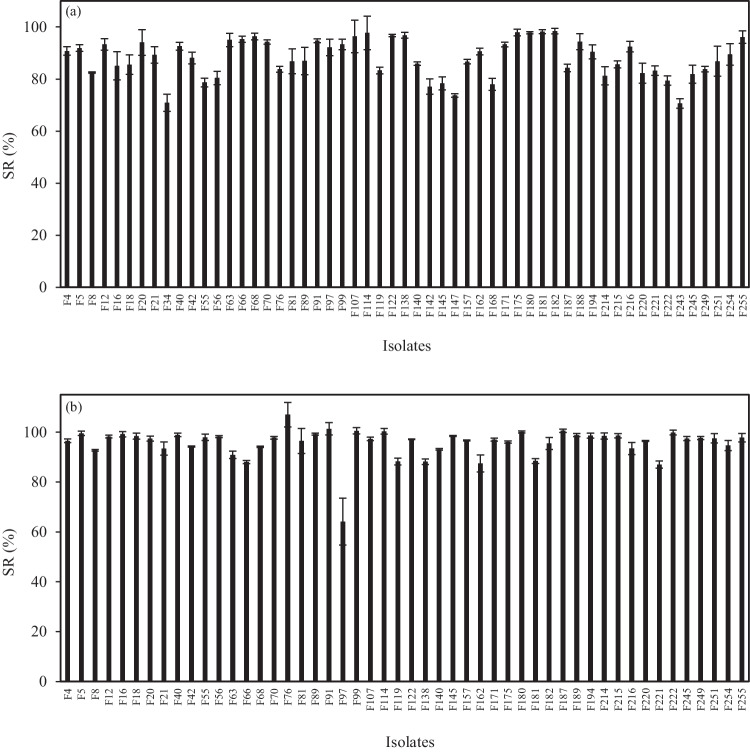
Survival rate (SR) of the 55 selected LAB isolates during in vitro tests under **a** simulated gastric phase (pH 2.5, 3h) and **b** simulated intestinal phase (1% bile salts, pH 8.0, 3 h) at 37 °C

A preliminary screening was carried out on isolates in order to determine their tolerance to low pH and the presence of bile salts. Out of the 93 isolates tested, 38 isolates were characterized as sensitive, since viable counts were recorded < 6 log cfu/mL after 3 h of incubation at pH 2.5. The remaining 55 isolates that demonstrated final population levels above that threshold were selected for further analysis (Fig. 2a). The majority of isolates’ viability loss was less pronounced, since over 50% of the isolates displayed resistant rates ranging from 83.75 to 98.39 (final counts > 8 log cfu/mL). Notably, five isolates, namely, F34, F142, F147, F168, and F243, exhibited a viability loss of more than 2 log cfu/mL and were excluded for further study.

Following the assessment of acid tolerance ability and based on the aforenoted results, the remaining 50 isolates were further evaluated. Figure 2b displays the observed survival rates of the tested isolates in the presence of 1% (w/v) bile salts. Even though in the present study a high percentage of bile salts was used, nearly all strains presented a resistant rate of around 90–100%. Specifically, 35 isolates showed a survival rate > 95% with growth capabilities exceeding 9 log cfu/mL in most cases. Viability loss for all isolates was detected no greater than 0.5 log cfu/mL. The only exception concerns isolate F97 which exhibited the least resistance (64.11% survival rate), which corresponded to an actual reduction of 3.8 log cfu/mL after 3 h of exposure. Consequently, 49 isolates were further investigated for their safety, as described below.

### Assessment of Safety Characteristics

#### Hemolytic Activity Evaluation

The characterization of LAB isolates as non-hemolytic is a crucial safety criterion when selecting a probiotic, ensuring that the selected strains are safe for human consumption. According to our results, no clearing zone (β-hemolysis) was found on blood agar in any of the 49 wild-type isolates examined. The majority of LAB (31 isolates) displayed a greenish halo when grown in Columbia blood agar during the test. Even though partial hemolytic activity (α-hemolysis) among LAB isolates of dairy origin is not uncommon [32, 33], the final selection was limited only to isolates which exhibited γ-hemolysis.

#### Antibiotic Susceptibility

The antibiotic susceptibility of selected non-hemolytic isolates was assessed according to the microbiological cut-off values as defined by the European Food Safety Authority (EFSA). Specifically, antibiotic evaluation was conducted on the isolates which had rod-like morphology (14 isolates). All LAB strains were characterized as sensitive to the commonly used antibiotics such as ampicillin, streptomycin, chloramphenicol, and erythromycin. Only three resistant phenotypes were observed in this experiment (Table 3). In specific, strain *Lcb. paracasei* F70 showed resistance to kanamycin, *Lpb. plantarum* isolate F194 showed resistance to both gentamycin and kanamycin, while *Lcb. paracasei* isolate F214 was found resistant to tetracycline.

**Table 3:** Probiotic features of selected LAB isolates

LAB isolates	Antibiotic resistance^1^	BSH activity^2^	Lysozyme resistance		α-Glucosidase
			Relative growth (%)	Inhibition (Δlog)^3^	Inhibition (%)
*Lpb. plantarum* F16	-	+	97.50 ± 1.12^bc^	0.20	4.15 ± 1.92^abc^
*Llb. fermentum* F55	Kanamycin	-	95.48 ± 0.86^bc^	0.36	0.93 ± 0.55^a^
*Lcb. parasacei* F70	-	+	97.21 ± 0.48^bc^	0.23	9.10 ± 2.25^de^
*Lpb. plantarum* F89	-	+	96.67 ± 1.16^bc^	0.28	5.36 ± 1.75^bcd^
*Lcb. rhamnosus* F107	-	+	102.82 ± 1.97^a^	−0.22	15.07 ± 0.25^f^
*Lcb. rhamnosus* F122	-	**+**	97.09 ± 0.28^bc^	0.24	18.91 ± 1.95^g^
*Lpb. plantarum* F162	-	++	99.05 ± 0.97^ab^	0.07	24.33 ± 2.02^h^
*Lpb. plantarum* F180	-	+	88.04 ± 0.46^d^	1.03	3.67 ± 2.00^abc^
*Lpb. plantarum* F194	Gentamycin, kanamycin	++	96.70 ± 1.65^bc^	0.28	3.38 ± 1.76^ab^
*Lcb. parasacei* F214	Tetracycline	+	92.96 ± 2.14^cd^	0.58	2.47 ± 2.16^ab^
*Lcb. parasacei* F216	-	+	96.80 ± 0.34^bc^	0.26	3.81 ± 0.46^abc^
*Lcb. parasacei* F220	-	**+**	96.85 ± 0.45^bc^	0.26	9.69 ± 2.67^e^
*Lpb. plantarum* F222	-	++	97.30 ± 1.15^bc^	0.21	7.71 ± 1.06^cde^
*Lpb. plantarum* F254	-	+	98.47 ± 0.84^ab^	0.13	4.94 ± 1.10^abc^
*L. rhamnosus* GG	-	+	94.70 ± 1.93^bc^	0.44	38.96 ± 2.52^i^

^a^Different superscript letters indicate significant differences (*p* < 0.05) within the columns

^b^No antibiotic resistance

^c^No precipitation to TDCA, ^+^precipitation, ^++^intense precipitation

^d^Inhibition (Δlog) = log_10_ (initial population) − log_10_ (final population)

### Genotypic Identification of Selected LAB Isolates

The results indicated that 18 isolates successfully underwent the preliminary GI tolerance assessment and met the safety criteria. Out of the 18 isolates, there were four cocci which were examined using KAA in order to indicate presumptive enterococci. All four isolates reacted positively to aesculin agar; hence, these isolates were excluded from molecular identification. Of note, *Enterococcus* spp. are not yet included in the qualified presumption of safety (QPS), a list indicating its suitability for food industry applications [18].

Sequencing of rRNA amplicons revealed mesophilic LAB, including species of *Lpb. plantarum*, *Lcb. paracasei*, *Lcb. rhamnosus*, and thermophilic *Limosilactobacillus fermentum*. For the majority of isolates, BLAST search resulted in identical hits of closely related species indicating the inability of the 16S rRNA gene to distinguish species within the *Lcb. casei* (*Lcb. casei*, *Lcb. paracasei*, and *Lcb. rhamnosus*) and *Lpb. plantarum* (*Lpb. plantarum*, *Lpb. paraplantarum*, and *Lpb. pentosus*) groups. Therefore, the phylogenetic affiliation of isolates was discriminated by species-specific PCR. In particular, specific multiplex PCR assays for the species identification within these groups were performed and the products were analyzed on a 2.5% agarose gel (Supplementary material Fig. S1). Overall, seven isolates (F16, F89, F162, F180, F194, F222, F254) were assigned to *Lpb. plantarum species* (319 bp), four isolates (F70, F214, F216, F220) were identified as *Lcb. paracasei* (253 bp), two isolates (F107, F122) were identified as *Lcb. rhamnosus*, and one was identified as *Llb. fermentum* (F55) (Table 3). The obtained sequences were deposited in GenBank and all accession numbers are cited as supplementary data (Supplementary material Table S1).

### Characterization of Isolates for Probiotic Properties

#### BSH Activity

In the present study, 13 out of 14 selected LAB strains exhibited bile salt hydrolase activity at different levels, displaying precipitation zones as halos around the colonies or presenting different colony morphology on MRS plates assayed supplemented with TDCA. More specifically, intense precipitation was determined for the isolates *Lpb. plantarum* F162, F194, and F222 (Table 3).

#### Lysozyme Resistance and Phenol Tolerance

All selected strains showed high tolerance at the concentration of lysozyme examined with maximum survival values of up to 96.69% after 2 h of exposure (Table 3). *Lpb. plantarum* strains presented elevated resistance to lysozyme between 88.4 and 99.5%, with a variation of 0.07–1.05 log reduction. In terms of the survival rate at 100 µg/mL of lysozyme, all strains presented a slight viable count decrease of 0.07–1.03 log cfu/mL. Strain *Lcb. rhamnosus* F107 was the most resistant to this treatment (*p* < 0.05), whereas only *Lpb. plantarum* F180 showed a significant degree of sensitivity (*p* < 0.05) among the tested group.

In Table 4, the ability of strains to withstand 0.4% phenol is demonstrated, indicating a moderate tolerance. All tested strains exhibit good viability, whereas eight out of 14 strains were even able to grow in the presence of 0.4% phenol. For the rest of the strains, a bacteriostatic effect was observed, since their numbers did not increase from the initial inoculum. In particular, *Lcb. rhamnosus* F122 and *Lpb. plantarum* F254 strains exhibited the greatest resistance to phenol treatment, demonstrating approximately a 0.5 log increase in growth. On the other hand, *Lpb. plantarum* F180, F194, and F222 strains were particularly sensitive, since their viability was considerably inhibited showing a reduction of 0.89, 1.45, and 0.71 log cfu/mL, respectively. Overall, the survival and growth of the examined LAB strains were slightly influenced by phenol over 24 h of incubation, suggesting that bacteria may survive throughout GI transit time.

**Table 4:** Phenol tolerance of selected LAB isolates

LAB isolates	Viable counts (log cfu/mL)		Growth (Δlog)^a^
	MRS + 0.4% phenol concentration		
	*T*_0_	*T*_24_	
*Lpb. plantarum* F16	6.91 ± 0.11	6.65 ± 0.18	−0.27
*Llb. fermentum* F55	7.01 ± 0.05	6.70 ± 0.14	−0.31
*Lcb. parasacei* F70	7.20 ± 0.13	6.89 ± 0.34	−0.31
*Lpb. plantarum* F89	7.40 ± 0.06	7.67 ± 0.25	0.27
*Lcb. rhamnosus* F107	6.72 ± 0.05	7.06 ± 0.02	0.34
*Lcb. rhamnosus* F122	7.27 ± 0.23	7.84 ± 0.13	0.57
*Lpb. plantarum* F162	6.64 ± 0.18	7.05 ± 0.08	0.41
*Lpb. plantarum* F180	7.61 ± 0.24	6.72 ± 0.24	−0.89
*Lpb. plantarum* F194	7.40 ± 0.28	5.95 ± 0.01	−1.45
*Lcb. parasacei* F214	7.18 ± 0.17	7.25 ± 0.44	0.07
*Lcb. parasacei* F216	7.16 ± 0.15	7.24 ± 0.43	0.08
*Lcb. parasacei* F220	7.21 ± 0.04	7.29 ± 0.24	0.08
*Lpb. plantarum* F222	6.80 ± 0.06	6.09 ± 0.14	−0.71
*Lpb. plantarum* F254	7.25 ± 0.07	7.76 ± 0.11	0.51

^a^Growth (Δlog) = log_10_ (final population) − log_10_ (initial population)

#### Antimicrobial Activity Profile

**Fig. 3:**
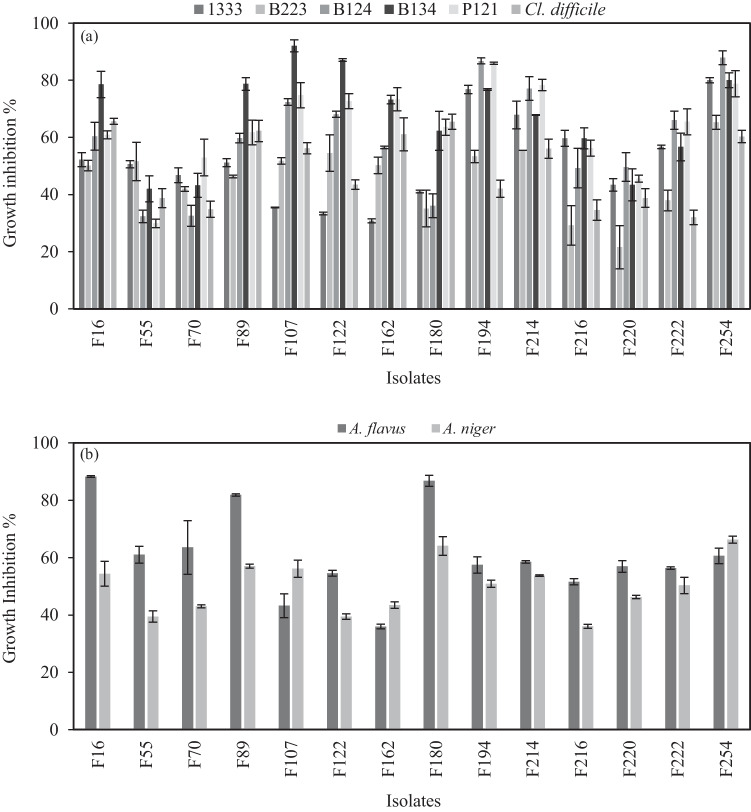
Antimicrobial activity of selected LAB strains. **a** Growth inhibition of CFSs is expressed compared to controls of pathogen targets: *Ps. aeruginosa* B223, *Staph. epidermidis* P121, *L. monocytogenes* P124, *Staph. aureus* B124, *Kl. oxytoca* P1333, and *Cl. difficile*. **b** Antagonistic activities against *A. flavus* and *A. niger*

The antibacterial activity of the 14 selected strains was tested against nine common food-related pathogen microorganisms. In the first part of this assay, bacterial CFSs with pH ranging from 3.72 to 3.87 were tested, highlighting substantial inhibitory action towards bacterial pathogens since no growth was observed (data not shown). Thus, in order to examine the nature of these activities, CFSs were neutralized to pH 6.5. The neutralization of CFSs resulted in an intensive decrease in growth inhibitory efficacy against all pathogen strain targets (Fig. 3a). All strains’ extracts exhibited a broad spectrum of antimicrobial activities displaying varying degrees of inhibition, speculating the action of proteinaceous compounds. In detail, in the CFSs of all *Lpb. plantarum* strains, high growth inhibitory activity was observed against *Staph. aureus* (56.67–80.01%), *Staph. epidermidis* (60.90–85.98%), and *L. monocytogenes* (35.08–87.98%). In most cases, Gram-negative bacteria were generally more pronounced than Gram positive. However, the CSF of strain F254 presented increased inhibition effect against *Ps. aeruginosa* and *Kl. oxytoca* (65.24 and 80% respectively). CFSs of *Lcb. rhamnosus* strains (F107, F122) were also able to inhibit *Staph. aureus* (89.09–92.03%), *L. monocytogenes* (73.8–87.09%), and *Cl. difficile* (43.44–56.18%) growth*. Llb. fermentum* F55 CFS resulted in weaker inhibitory ability, reaching levels of 29.89 to 51.58% of growth decrease. The supernatants of *Lcb. paracasei* (F70, F214, F216, F220) cultures presented moderate activity. Among them, isolate F214 proved to have the most effective antagonist activity against all pathogen targets, whereas isolate F70 displayed the weakest action. Likewise, all LAB CFSs exhibited moderate growth inhibitory activity against *Kl. oxytoca* (30.79–80.01%).

In the second part of this experiment, a dual culture assay was undertaken to evaluate the effects of LAB strains on fungal growth, as displayed in Fig. 3b. The presence of all LAB strains greatly inhibited the growth of both *A. flavus* and *A. niger*, whereas *P. expansum* proved to be the least resistant target since no mycelial growth was observed in any of the co-cultures. Strains of *Lpb. plantarum* exhibited different levels of antagonism. Specifically, 35.98–88.26% inhibition was observed against *A. flavus* and 43.43–66.27% inhibition against *A. niger*. *Lcb. paracasei* strains demonstrated also comparable activities, reaching average values of 57.63% for *A. flavus* and 44.74% for *A. niger*. *Llb. fermentum* inhibited mycelial growth to the extent of 39.46–61.01%. Comparable degrees of inhibition were also recorded for both fungi, ranging to an average of 49.67% and 46.99% for strains F107 and F122, respectively.

#### Inhibition of α-Glucosidase

The inhibitory activities of the wild-type LAB CFSs against α-glucosidase ranged from 0.93 to 24.33%, with the highest value (*p* < 0.05) recorded in *Lpb. plantarum* F162 CFS (Table 3). Despite exhibiting significantly lower values (*p* < 0.05) compared to CFS of the reference GG strain (38.96%), the α-glucosidase inhibition of *Lpb. plantarum* F162 and *Lcb. rhamnosus* F107 and F122 strains demonstrated promising attributes.

#### Cholesterol Assimilation

**Fig. 4:**
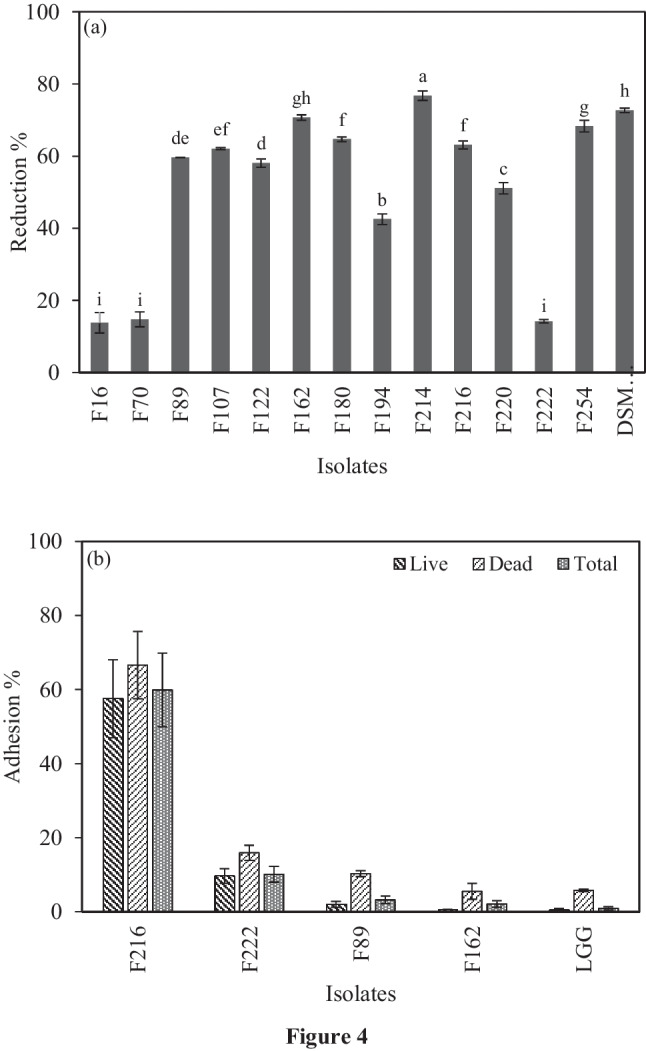
**a** Cholesterol reduction (%) by LAB strains in fermentation medium. **b** Cholesterol adhesion (%) on live, dead, and total LAB cells, as assessed by flow cytometry assay. Different letters in superscript represent significant differences among isolates (*p* < 0.05)

The ability of LAB strains to assimilate cholesterol was investigated and the results are shown in Fig. 4a. All strains were able to reduce cholesterol levels in the fermentation medium after 24 h of incubation. The cholesterol removal varied among the strains and ranged from 13.79 to 76.73%. Among the tested strains, *Lcb. paracasei* F214 showed the highest removal activity (76.73%) (*p* < 0.05). In addition, *Lpb. plantarum* strains F162 and F254 display activities similar to the reference strain (70.70, 68.29, and 72.69% respectively), followed by *Lcb. rhamnosus* F107, *Lpb. plantarum* F180, and *Lcb. paracasei* F216 (62.06–64.68%). The strains *Lpb. plantarum* F89, *Lcb. rhamnosus* F122, *Lpb. plantarum* F194, and *Lcb. paracasei* F220 exhibited moderate cholesterol assimilation (42.51–59.65%), whereas the lowest values were observed for the *Lpb. plantarum* F16, *Lcb. paracasei* F70, and *Lpb. plantarum* F222 strains (13.79–14.75%).

In addition, the results of the cholesterol adhesion assay on both live and dead, as well as on the total cell population, revealed that ten out the 14 strains showed insignificant or very low affinity for cholesterol (< 0.5%); in accordance with previous results for *Lpb. plantarum* strains [29]. However, the remaining four strains showed moderate to high adhesion potential (Fig. 4b); interestingly, one strain, namely, F216 belonging to *Lcb. paracasei*, exhibited a very high adhesion potential in both live and dead cells (57.5% on live cells, 66.6% on dead cells, and 59.9% on total cell population).

#### In Vitro Adherence to Epithelial Cells

The potential of the 14 indigenous LAB recovered from Feta-type cheese to colonize intestinal epithelial Caco-2 cell lines was also evaluated and is presented in Table 5. Strains were classified into four groups based on the percentage of adhesion. Group 1 (% adhesion of 0.5–3.5) consisted of three *Lpb. plantarum* strains (F162, F180, and F194) that showed insignificant or very low adhesion; group 2 (% adhesion of 3.5–5.5) consisted of three strains with low adhesion (*Llb. fermentum* F55,* Lcb. paracasei* F214, and *Lpb. plantarum* F220) including the reference GG strain. Five strains showed adhesion in the range of 5.5–10.0% (*Lcb. paracasei* F70 and F216, *Lcb*. *rhamnosus* F107 and F122, and *Lpb. plantarum* F222), while three *Lpb. plantarum* strains (F16, F254, and F89) exhibited substantial adhesion (> 10%), with F16 and F254 displaying the maximum adhesion levels, 15.76% and 14.5%, respectively.

**Table 5:** Bacterial adhesion to Caco-2 monolayer cells in vitro

LAB isolates	% attachment to Caco-2	Range
*Lpb. plantarum* F16	15.76 ± 2.08^g^	> 10.0
*Lpb. plantarum* F254	14.57 ± 0.76^g^	
*Lpb. plantarum* F89	11.16 ± 0.55^f^	
*Lcb. paracasei* F216	9.32 ± 1.55^ef^	5.5–10.0
*Lpb. plantarum* F222	9.24 ± 0.96^ef^	
*Lcb. paracasei* F70	7.65 ± 0.61^de^	
*Lcb. rhamnosus* F107	6.67 ± 0.25^cde^	
*Lcb. rhamnosus* F122	5.98 ± 0.24^cde^	
*Lcb. paracasei* F214	5.45 ± 0.54^cd^	3.5–5.5
*L. rhamnosus* GG	5.33 ± 0.60^bcd^	
*Llb. fermentum* F55	4.75 ± 1.12^bcd^	
*Lcb. paracasei* F220	4.49 ± 0.77^bc^	
*Lpb. plantarum* F180	3.44 ± 1.22^abc^	0.5–3.5
*Lpb. plantarum* F162	2.23 ± 0.89^ab^	
*Lpb. plantarum* F194	0.73 ± 0.29^a^	

Different superscript letters indicate significant differences (*p* < 0.05)

### Milk Acidification Assessment

**Fig. 5:**
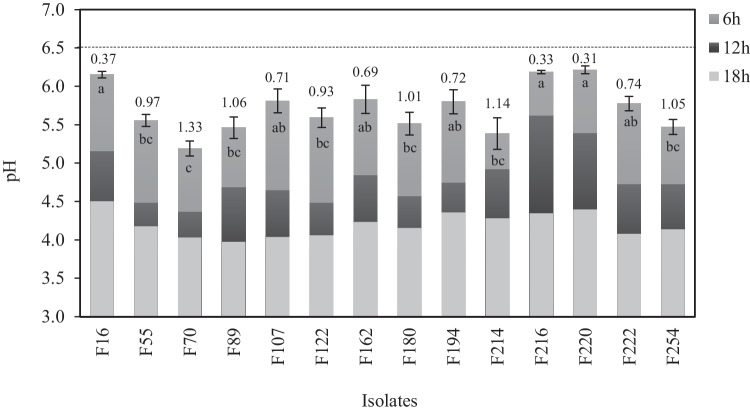
Skim milk acidification ability of selected LAB strains. Values above bars indicate the ΔpH drop between inoculation time (initial pH 6.5) and the first 6 h of acidification process. Different letters in superscript represent significant variances among isolates (*p* < 0.05)

The fermentation performance potential, of single-strain inoculation in skim milk, was also assessed in this study. The pH values of the milk were highly affected by fermentation time, reaching levels of 6.32 and 3.98 at 6 and 18 h, respectively (Fig. 5). The majority of strains displayed a medium acidification dynamic within the first 6 h. The highest decrease rates were achieved by strains F55, F70, F89, F180, F214, and F254 (ΔpH 0.97–1.33), within 6–12 h of fermentation. For the rest of the strains, coagulation was also observed after 24 h of fermentation (data not shown). *Lcb. paracasei* F70 is outlined as the faster acidifying strain, which reduced the pH value of skim milk within 6 h to 5.19, fulfilling the abovementioned criterion. It is worth noting that the *Lcb. paracasei* group conferred the higher strain-related activity, since strains F216 and F220 were characterized as the weakest acidifiers of milk (ΔpH 0.33 and 0.31, respectively, at 6 h). Even so, over the course of 18 h of fermentation, pH levels were measured to be relatively near to those of the most potent acidifiers.

## Discussion

The present work provides, for the first time, insights into the microbial communities of the traditionally manufactured Kefalonian Feta-type cheese. The microbial groups identified in the cheese samples included those typically observed in white-brined cheeses. Presumptive LAB counts were predominant in all cheese samples; these results are consistent with previous observations regarding traditional white-brine cheeses. Nevertheless, in this study, levels were notably higher than typically reported for Greek white-brine cheeses [2, 11]. Both NSLAB and enterococci diversity constitute a crucial part of traditional white-brine cheeses’ microbiota, having a significant role in flavor development [34, 35]. Yeasts are often a part of the secondary microflora of white-brine cheeses [36]. The presence of *Enterobacteriaceae*, coliforms, pseudomonads, and micrococci usually indicates suboptimal hygiene conditions or manufacturing practices [10]. This is, however, not uncommon in traditional small-scale production techniques.

Initially, a preliminary GI tolerance assessment was performed, targeting to investigate the resistance of the 93 LAB isolates against acidic conditions and bile salts. Acid and bile tolerance is considered the initial prerequisite for in vitro evaluation of whether a microorganism has the potential to play a probiotic role and confer health benefits on the host. When compared to similar research efforts, the results of the present study are noteworthy [9, 37]. However, it is important to note that the protection provided by the food matrix significantly enhances probiotic viability during the acidic conditions of GI transit [38, 39]. In general, the strains in this study exhibited greater robustness in the presence of bile salts than in the acidic environment. Bile salts are considered antimicrobial agents, compromising the bacterial membrane integrity of intestinal microbiota through various means [40, 41]. Their presence in the upper small intestine is a critical challenge, as microorganisms must reach the colon in a viable state. In addition, intestinal colonization requires the presence of intrinsic resistance mechanisms to bile salts [42]. Advances in this area have identified novel functional genes, associated with bile salt tolerance, in species such as *Lpb. plantarum*, *Lcb. paracasei*, and *Llb. fermentum*. A potential relationship between strain origin, phenotype, and bile salt resistance–related genes has also been recently reported [43]. Our findings align with comparable studies, highlighting advancements in resistance. It should be acknowledged, however, that bile acids may still induce disruption of cell membranes [44].

In the subsequent experiments, the isolated LAB strains were further evaluated for their safety (hemolytic activity and antibiotic susceptibility). The assessment of hemolytic activity elicits an important criterion for selecting probiotics intended to be used in fermented food products as adjunct or probiotic cultures. Even though the isolated bacteria have generally recognized as safe (GRAS) or qualified presumption of safety (QPS) status, the EFSA strongly recommends evaluating this characteristic before considering food applications [18]. The assessment of antibiotic resistance in potential probiotic strains is also closely linked to food safety issues. Lactobacilli species have historically been regarded as safe; however, food-born isolates have also been recognized as potential reservoirs of antibiotic resistance genes [45]. Pavli et al*.* [46] detected isolates of *Lpb. plantarum* resistant to gentamycin, kanamycin, and tetracycline. In general, lactobacilli are recognized to be naturally resistant to kanamycin, albeit with species- or strain-related variations [9]. This intrinsic resistance to aminoglycosides (kanamycin, gentamycin, and streptomycin) is chromosomically encoded and thus not transmissible [47]. On the other hand, a significant risk of resistance transfer to pathogenic and commensal gut bacteria is associated with acquired resistance. Concerning tetracycline, key resistance genes prevalent in lactobacilli were early reported in strains isolated from cheese and other dairy products [48]. Most of these genes are associated with transmissible plasmids or transposons resulting in rapid spread among bacteria [49, 50]. In cases, like that of the isolate *Lpb. plantarum* F214, the use of molecular technics to unravel the genotype of such virulence traits is crucial, ensuring their potential application in food systems. While contemporary lactobacilli whole-genome sequencing data offer new insights into drug resistances [51, 52], our study employed a phenotypic approach to assess the antibiotic resistance profile of the LAB strains.

The 18 isolates, resulted by the preliminary screening and safety characterization, were identified through molecular techniques. Subsequently, the 14 rod-like morphology isolates were selected to be further investigated for their probiotic attributes. BSH activity, due to the advantageous role of bile salts in various metabolic processes [53, 54], is another selection criterion illustrating for potential probiotic bacteria. Phylogenetic analyses have revealed a substantial number of distinct, highly conserved *bsh* genes highly distributed in lactobacilli niche [54]. In addition, several reports demonstrated inter-strain patterns in these species linked to the substrate specificity of BSH enzymes which might explain our findings regarding the BSH-negative isolates [19, 33, 55]. The phenotypic responses, however, in these qualitative assays are not always correlated with a BSH-active genotype [56–58]. In line to our findings, the claim that BSH decreases bile acid toxicity is herein also doubtfully supported [59]. Foley et al*.* [44] indicated that *Lactobacillus* inhibition from deconjugated BAs remained unaltered by the presence or absence of BSHs. Current research has revealed that distinct *bsh* genes of *Lpb. plantarum* differentially affect the growth and adhesion of microorganisms under bile acids/salts, suggesting stimulation or limitation of cell proliferation in the presence of bile acids [60]. Nevertheless, whether harboring a *bsh* gene is a preferable strain trait, enhancing the challenge of safety aspects of probiotics remains unclear [44, 61]. Despite its prevalence, BSH function is postulated as one of the most contradictory topics since accumulated data demonstrate controversies associated with elevated levels of deconjugated bile acids [62, 63]. Although the mechanisms of action of BSH-active probiotic bacteria require further support, they still provide valuable information when selecting a strain. These results may be of particular interest, taking into consideration that LAB strains are routinely used in the production of dairy products.

The susceptibility of bacteria to lysozyme is a promising criterion for the selection of LAB as probiotic candidates. Lysozyme are antimicrobial enzymes found in saliva or released by the GI mucosa that disrupt the glycan backbone in the peptidoglycan polymer present in bacterial cell walls. The findings of the present study support the results of previous works where LAB strains exhibited varying degrees of lysozyme resistance [64, 65]. In line with this, the response of lactobacilli to lysozyme has been attributed to inter- and intra-species specificities [66, 67]. The susceptibility of isolates to severe GI conditions was further evaluated. Resistance to phenol is considered an essential quality test for determining the viability of bacteria. Harmful metabolites generated during digestion, such as several aromatic amino acids, can be deaminated by gut bacteria producing toxic catabolic products like phenol [68]. Although these isolated species belonged to physiologically closely related species, different levels of sensitivity to phenol were detected. Strain specificity towards LAB phenol resistance is commonly recorded along literature [65, 69]. Specifically, for *Lpb. plantarum* strains, variability in phenol tolerance has been reported in the past, indicating diverse bacteriostatic effects [70, 71].

The ability of LAB to counteract gut or intestinal pathogens by secreting antimicrobial compounds (organic acids, hydrogen peroxide, bacteriocins) and protecting the host is an enviable attribute for probiotic potentiality [72–75]. The observations in the current work align with previous reports, indicating that potential probiotic indigenous lactobacilli can display a broad inhibition spectrum against foodborne pathogens [22, 76, 77]. In addition, the results are consistent with previous research, demonstrating the ability of probiotic strains to inhibit the growth of specific fungi [78, 79]. Variability in antifungal properties has earlier been correlated with strain specificity of *Lpb. plantarum* [23]. Recent research has revealed that high levels of organic acid synthesis correlate favorably with antifungal activity in *Lpb. plantarum* strains [80]. On the other hand, peptides, produced by LAB, have been identified as important antifungal compounds, often disrupting fungal membrane integrity and damaging the mycelia [81, 82]. Potential probiotic strains of *Lcb. rhamnosus* have also been found to possess antifungal properties [22, 83], indicating that LAB can serve as antifungal agents [84]. Overall, our findings emphasize strain- rather than species-dependent antimicrobial attributes. Often enough, no antimicrobial activity of newly isolated LAB strains is documented [11, 12]. Therefore, the number of research screenings of LAB from diverse sources to unravel probiotic strains is constantly increasing. Typically, the generation of antimicrobial compounds in vitro alone cannot provide accurate results about the behavior of probiotics in vivo. The findings of the current or similar works indicate only the antagonistic potential of the recovered isolates. Furthermore, antimicrobial activity is an immensely complicated mechanism, demonstrating exceedingly high strain specificity of the pathogen target, necessitating independent studies for validation.

The CFSs of the 14 wild-type LAB strains were also tested for α-glucosidase inhibitory activity and were compared to GG strain, a commercial probiotic strain associated with antidiabetic properties [85]. α-Glucosidase, a digestive enzyme, located in the brush border of the small intestine [24], catalyzes the hydrolysis of 1,4-α-glycosidic bonds in complex carbohydrates and oligosaccharides leading to the release of glucose [86]. The inhibition of α-glucosidase activity has been reported to reduce postprandial blood glucose levels and glucose absorption from the digestive tract [24, 86, 87], thus making it a useful tool for controlling blood glucose levels in patients with type 2 diabetes (T2D). Competitive inhibitors of α-glucosidase, such as miglitol, voglibose, and acarbose, are commonly used as antidiabetic agents in the case of T2D [88]. However, their use has been associated with several GI side effects such as diarrhea and flatulence [24, 89]. Additionally, several studies have reported the ability of LAB to inhibit the activity of α-glucosidase either through secreted polysaccharides [24, 90] or peptides [91] produced by the bacteria. Consequently, to circumvent negative side effects, the oral supplementation of LAB strains, able to inhibit α-glucosidase, has been suggested as a promising alternative to antidiabetic drugs [22].

The cholesterol assimilation capability of probiotics and the mechanisms involved in cholesterol reduction have recently gained increased attention. High levels of serum cholesterol are widely regarded as a risk factor for cardiovascular diseases. Thus, the food and pharmaceutical industry is seeking for safer and more effective alternatives for cholesterol reduction, given that drugs may lead to several side effects. Various mechanisms underlying the cholesterol-lowering benefits of probiotics have been studied in vitro. One of the proposed mechanisms is the enzymatic deconjugation of bile acids by probiotic bile-salt hydrolase [56]. The ability of probiotics to bind cholesterol in the small intestine has also been linked to their hypocholesterolemic impact, and it appears to be affected by their growth phase [92]. Furthermore, during the growth phase, probiotics assimilate cholesterol by incorporating it into the cellular membranes; this mechanism alters the fatty acid composition of cells, enhancing membrane stability and subsequently cellular resistance to lysis [93]. In addition, probiotics may contribute to the transformation of cholesterol to coprostanol by cholesterol dehydrogenase/isomerase, which catalyzes the transformation of cholesterol to cholest-4-en-3-one, an intermediate co-factor in the conversion of cholesterol to coprostanol [94]. Previous studies suggest a considerable diversity in the cholesterol-lowering effect among different species or even strains [95, 96]. In accordance with our results, Albano et al*.* [96] reported that strains of *Lcb. paracasei* and *Lpb. plantarum*, isolated from traditional Italian cheese, reduced cholesterol levels by more than 55%. Similarly, Miremadi et al*.* [97] reported that in vitro cholesterol reduction of about 50% has been noted by *Lcb. paracasei* and *Lpb. plantarum* strains of human origin. Furthermore, it seems that there is a significant correlation between the in vitro ability of lactobacilli to digest cholesterol and their hypocholesterolemic impact in vivo [98, 99]. Indeed, Ding et al*.* [98] observed that the administration of a *Lpb. plantarum* strain, exhibiting in vitro a 73.3% cholesterol removal ability, significantly reduced cholesterol levels in rats following a high cholesterol diet. Although the tested strains in our study demonstrated a high potential for decreasing cholesterol levels, further research is required to investigate their cholesterol-lowering activity in vivo.

The ability of LAB to attach to intestinal surfaces and subsequently colonize the gut are common criteria for probiotic strain selection. Research has shown that lactobacilli and other probiotics can interact with differentiated Caco-2 cells that are used as a cellular model of the intestinal epithelium [100, 101]. Thus, this cell line is frequently employed as an in vitro model for studying the absorption and transport of nutrients and drugs across the intestinal epithelium. Notably, recent studies suggest that short-term adherence and temporary colonization are sufficient for favorable probiotic activities in the host [102]. The adhesion potential of the strains, in the present study, varied widely and was not correlated with the species; these results are in accordance with previous studies [103, 104]. The cell-based assay, utilized in this work, serves as a preliminary tool for discriminating between strong and weak adherence strains. Similar results have been obtained in several studies analyzing the adhesion capacity of probiotic bacterial strains to differentiated Caco-2 cells [105, 106]. Indigenous lactobacilli characterized by Zoumpopoulou et al. [59] exhibited limited adhesion ability (up to 6%). More recently, novel strains with significant adhesion ability (up to 13.64%) to cells have been identified [107]. Inter-strain differences appear to be prevalent for this attribute [108]. The restricted adhesion ability observed for the GG strain is also consistent with previous works [104, 109]. Overall, conducting a comparative assessment with literature data remains challenging, underscoring the constraints imposed by diverse methodologies employed in different studies.

Additionally, it is noteworthy that several of the newly isolated strains have specific technological relevance for dairy applications, such as milk acidification or clot formation, which hold significant importance in the field of dairy technology [110]. As commonly defined, a starter culture is capable of lowering the pH value to about 5.3 within 6 h at 30–37 °C [111]. Still, strains with moderate acidifying capacities, but notable other functional traits, can also be used as adjunct cultures and combined with stronger acidifiers to create novel starter cultures composites.

This novel pool/collection of lactic acid bacterial strains from Kefalonian Feta-type cheeses has revealed a previously unrecognized source of 14 potential probiotic strains. Among these, three *Lpb. plantarum* strains, namely, F16, F254, and F89, exhibited high epithelial adhesion ability, while *Lcb. paracasei* F214 demonstrated the most significant cholesterol reduction, followed by the strains *Lpb. plantarum* F162 and F254. Similarly, *Lpb. plantarum* F162 displayed the highest α-glucosidase inhibition, whereas *Lcb. paracasei* F216 showed the highest cholesterol adhesion, followed by the *Lpb. plantarum* strains F222, F89, and F162. Notably, *Lcb. paracasei* F70 was characterized as a defined strain starter. All tested strains in vitro appeared to be safe and exhibited promising characteristics related to probiotic traits.

## Conclusions

The screening in this work highlighted several prominent isolates, each exhibiting multifunctional potential. It is well known that traditional cheeses have distinct characteristics and organoleptic attributes due to their autochthonous microbiota. The selection of native strains might facilitate the standardization of their manufacturing process, ensuring quality and safety, whereas possible certifications, including PDO and PGI, may be proposed to promote their production. Additionally, culinary tourism, a newly introduced concept, focuses on exploring local products distinguished by unique flavors, cultural and historical significance, and distinctive production techniques. In this respect, the identified lactic acid bacteria can signify the signature of a specific product within an authenticity concept. In summary, based on the probiotic characterization results, it can be concluded that the most promising strains were *Lpb. plantarum* F89, F162, and F254 and *Lcb. paracasei* F214 and F216, while *Lcb. paracasei* F70 showed also potential as a defined strain starter. These promising strains could be explored in various fields, including the food industry, biotechnology, and nutrition, contributing not only to biodiversity preservation but also to the development of novel food formulations within the context of the bioeconomy and in alignment with the Sustainable Development Goals (SDGs).

## Supplementary Information

Below is the link to the electronic supplementary material.

Supplementary file1 (DOCX 399 KB)

## Data Availability

All data generated or analyzed during this study are included in this article. Any additional information can be made available, upon reasonable request, by the corresponding author.
