# Supplementary material for: Novel Probiotic Candidates in Artisanal Feta-Type Kefalonian Cheese: Unveiling a Still-Undisclosed Biodiversity
Source: Probiotics Antimicrob Proteins. 2024 Mar 13;17(5):2909–27. doi: 10.1007/s12602-024-10239-x (PMC12532650; doi:10.1007/s12602-024-10239-x)
Supplement: Supplementary file 1 — Supplementary file1 (DOCX 399 KB) [file 12602_2024_10239_MOESM1_ESM.docx]

**Supplementary Files**

**Table S1** GenBank accession numbers of studied LAB

| **Strain** | ***16S ribosomal RNA gene*, partial sequence Acc. No** |
| --- | --- |
| **F16** | OQ845785 |
| **F55** | OQ845786 |
| **F70** | OQ845787 |
| **F89** | OQ845788 |
| **F107** | OQ845789 |
| **F122** | OQ845790 |
| **F162** | OQ835714 |
| **F180** | OQ845791 |
| **F194** | OQ835708 |
| **F214** | OQ835709 |
| **F216** | OQ835710 |
| **F220** | OQ835711 |
| **F222** | OQ835712 |
| **F254** | OQ835713 |


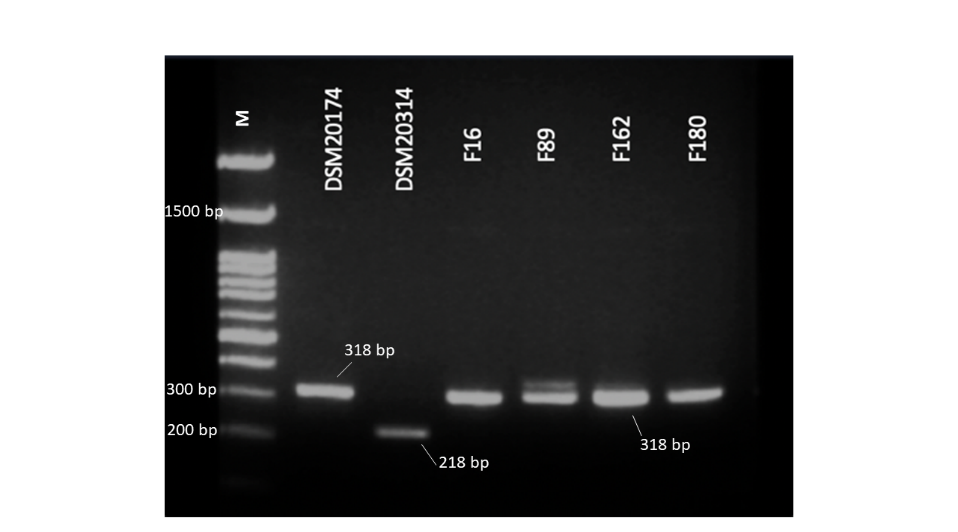
**
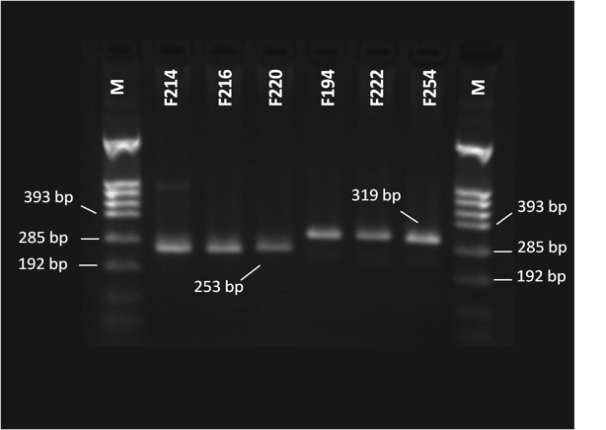
**

**(a)**

**(b)**

**Fig. S1** Agarose gel electrophoresis of **(a)** *Lpb. plantarum* group-specific multiplex PCR products (DSM 20174 corresponds to *Lactiplantibacillus plantarum* and DSM20314 to *Lactiplantibacillus pentosus*) **(b)** *Lcb. paracasei* and *Lpb. plantarum* group-specific multiplex PCR products (lane M indicate DNA size marker).
